# Patient Satisfaction with Private Genetic Counselling for Familial Cancer in Western Australia: A Prospective Audit

**DOI:** 10.31557/APJCP.2021.22.10.3253

**Published:** 2021-10

**Authors:** Charmi N Perera, Sarah O’Sullivan, Nicholas Pachter, Jason Jit-Sun Tan, Paul A Cohen

**Affiliations:** 1 *School of Medicine, University of Notre Dame Australia, Fremantle Campus, Fremantle, Western Australia, Australia. *; 2 *Women Centre, West Leederville, Western Australia, Australia. *; 3 *Genetic Services of Western Australia, King Edward Memorial Hospital, Subiaco, Western Australia, Australia. *; 4 *Division of Pharmacology and Division of Paediatrics and Child Health, University of Western Australia Faculty of Medicine Dentistry and Health Sciences, Crawley, Western Australia, Australia. *; 5 *Division of Obstetrics and Gynaecology Faculty of Health and Medical Sciences,University of Western Australia, Crawley, Western Australia, Australia. *; 6 *Division of Gynaecological Oncology, St John of God Subiaco Hospital, Subiaco, Western Australia, Australia. *; 7 *Institute for Health Research, University of Notre Dame Australia, Fremantle Campus, Fremantle, Western Australia, Australia. *

**Keywords:** Genetic counselling, genetic services, practice models, program evaluation and private healthcare

## Abstract

**Background:**

The rapid increase in demand for cancer genetic testing in Australia led to the establishment of private Familial Cancer Clinics (FCCs) as alternatives to public sector FCCs. Australian studies conducted in the public sector have shown high patient satisfaction with genetic counselling. No study has investigated patient satisfaction with genetic counselling in the private sector in Australia. Our aim was to assess patient satisfaction with genetic counselling for familial cancer within the private healthcare sector of Western Australia.

**Materials and Methods:**

Questionnaires were given to all eligible patients after their first genetic counselling appointment, consisting of the 12-item Satisfaction with Genetic Counselling Scale and an added question regarding the perceived value for the financial cost. Outcomes assessed included instrumental satisfaction, affective satisfaction, procedural satisfaction and perceived value for financial cost. Participants scored the representative questions from one to four (unsatisfied - highly satisfied).

**Results:**

Two hundred and twenty patients were given the questionnaire, 75 questionnaires were returned (response rate 34%), and 73 were appropriately completed and analysed. Overall, seventy (96%) participants were highly satisfied with the genetic counsellor’s explanation; seventy-four (98%) were highly satisfied/satisfied with the reassurance provided. Sixty-eight participants (93%) were highly satisfied/satisfied with the help received. Seventy-two (99%) participants had their expectations met and sixty-nine (95%) participants were highly satisfied with the service. Sixty-eight (93%) participants were highly satisfied/satisfied with the cost of private genetic counselling. Sixty-one (83.6%) proceeded to genetic testing.

**Conclusions:**

Private genetic counselling was considered highly satisfactory, and the cost considered acceptable by most participants.

## Introduction

The aim of cancer genetic counselling is to identify individuals at increased risk of cancer and provide information and support regarding their risks and risk management options, in order to reduce morbidity and mortality (Holloway et al., 2004). While demand for Familial Cancer Clinic (FCC) services has been high for more than a decade, in 2013 the so-called “Angelina Jolie Effect” led to significantly increased and sustained interest in, and demand for, FCC services (James et al., 2013). As a consequence, the demand for FCC services considerably outweighed the resources that were available to meet the demand (Evans et al., 2014). In part, this situation prompted the establishment of alternatives to traditional public sector FCC services in Australia.

In Australia, genetic counselling for familial/hereditary cancer is primarily available through FCCs that operate within the public healthcare sector. Public FCCs have stringent eligibility criteria for genetic counselling and genetic testing, meaning that services are not available to everyone who wishes to access them. For those who are eligible, costs are covered by Medicare and/or the FCC/hospital, however there can be significant waiting times for appointments and test results. 

Private FCCs are now operating in most Australian states. In recognition of the increasing trend towards private genetic counselling in Australia, practice guidelines have been developed (Sane et al., 2015; Collis et al., 2018). 

In early 2015, genetic counselling and testing for familial cancer became available in the private healthcare sector of Western Australia (WA). This represented a significant change to the provision of familial cancer genetic counselling in the state, which was previously available exclusively through Genetic Services of Western Australia (GSWA) – a single, state-wide clinical genetics service provided by the public healthcare sector. 

WA patients who are eligible for public FCC services now have the option to be seen at GSWA or privately. Almost all services provided in the public sector at GSWA are paid for by Medicare and are free to patients. However, waiting times for genetic counselling appointments and testing can be significantly longer in the public system compared to the private sector. Hence patients may elect to see a genetic counsellor (GC) in private and self-pay for genetic counselling and testing to reduce waiting times. These costs are currently not reimbursed by health insurance funds in Australia. The importance of reduced waiting times is increasing, as treatment decisions may be affected by variant status. For example, targeted therapy with Poly-(ADP)-ribose polymerase inhibitors has been shown to improve progression-free survival in BRCA pathogenic variant-positive ovarian cancer in combination with, and following, chemotherapy for platinum sensitive disease and as a single agent in recurrent disease (Friedlander et al., 2018; Moore et al., 2018; Coleman et al., 2019). The private FCC also provides WA patients who are not eligible for public FCC services with access to genetic counselling and testing. Access to the public FCC is determined by resource availability and does not consider patient preferences. In general, diagnostic genetic testing is only offered to individuals with a personal history of cancer and a >10% pre-test probability of carrying a likely/pathogenic variant according to genetic risk assessment instruments, such as The Breast and Ovarian Analysis of Disease Incidence and Carrier Estimation Algorithm (BOADICEA) and the Manchester Score for hereditary breast/ovarian cancer (Evans, 2004; Lee et al., 2019). Those who have no personal history of cancer but are at increased risk due to their family history are usually ineligible for public genetic testing, unless a likely/pathogenic variant has previously been identified in the family. Therefore, patients wishing to access genetic counselling/testing who did not meet eligibility criteria for the public FCC were left without an alternative, local option prior to 2015. These patients now have the opportunity to proceed with genetic testing in the private sector, following discussion of the advantages, limitations and implications of testing with a GC.

Genetic testing can be accessed through some general practitioners or via companies that offer direct-to-consumer testing, but often these options may not provide the opportunity for adequate pre-test counselling to enable informed consent, and/or for individuals to discuss their results and implications with a GC. This could lead to false reassurance or undue anxiety especially if clinically significant findings are unanticipated (Roberts and Ostergren, 2013). Concerns regarding direct-to-consumer testing include patients not fully comprehending the risk information they receive (Frueh et al., 2011).

There is an increasing focus being placed on patient-reported satisfaction with their medical care (Epstein and Street, 2011). As healthcare funding transitions to a value-based model, the ability to demonstrate the value of genetic counselling through outcomes research will be essential. A 2017 rapid systematic review of outcome studies in genetic counselling identified only five studies that had investigated patient satisfaction with genetic counselling or satisfaction with decision-making (Madlensky et al., 2017).Two of these five studies were conducted in non-cancer populations (Hunter et al., 2005; Austin and Honer, 2008), two studies were in women at risk for breast cancer (Burke et al., 2000; Green et al., 2004), and one study assessed low rates of African American participation in genetic counselling and testing for pathogenic variants in BRCA1/2 (Halbert et al., 2012). Studies conducted in public FCCs in Australia have largely shown high patient satisfaction with genetic counselling including a 2005 study undertaken at GSWA in 122 participants, 24% of whom had attended the FCC (Duric et al., 2003; Davey et al., 2005). To our knowledge, no study has specifically addressed patient satisfaction with private genetic counselling for familial cancer in Australia. The objective of the current study was therefore to assess the satisfaction of patients with genetic counselling in the private healthcare sector of WA, in order to identify any unmet needs and to improve the delivery of care to these patients. 

## Materials and Methods

*Participants*

Participants were new patients who presented for genetic counselling and possibly testing for familial cancer at a solo private practice. Patients were eligible to participate if they were newly referred for genetic counselling because of a personal and/or family history suggestive of a hereditary cancer syndrome. Eligible patients were invited to participate in the study following their initial genetic counselling appointment. 

*Instrumentation*

The study design consisted of a questionnaire that assessed patient satisfaction via an amended version of the 12-item “Satisfaction with Genetic Counselling Scale” (SCS) (Shiloh et al., 1990). An additional question was included to assess participants perceived financial satisfaction – “Do you feel that private genetic counselling provided value for the financial cost involved?”. Participants were also given the opportunity to provide any general comments in an open text box. The patients were asked about their demographics, personal and/or family history of cancer, source of referral and whether they proceeded with genetic testing. The questionnaire (supplemental online file) was completed by participants within one month of their initial genetic counselling appointment. The SCS comprised three sub-scales that reflect three dimensions of satisfaction: 

1. Instrumental satisfaction: this component is the extent to which the respondent evaluates the health professional as having the required skills and provides appropriate treatment and reassurance.

2. Affective satisfaction: relates to the evaluation of the health professional’s behavior toward the client as a person showing interest and care. 

3. Procedural satisfaction: addresses the administrative components of the service.

Participants were asked to evaluate the following: explanation of the condition, reassurance provided by the GC, listening skills, dedication, understanding, satisfaction with help received and whether the services matched the participant’s expectations. Participants gave each component a score from one - four, where a score of one is considered unsatisfactory and a score of four is considered highly satisfactory.

There were also three individual items that specifically assessed general satisfaction (two items) and satisfaction with the information provided (one item). For each item, participants indicated their level of satisfaction using a Likert scale from one (low) to four (high). 

*Procedure*

Between July 1, 2016, and July 31, 2019, all 220 patients attending for their first genetic counselling appointment were invited to participate in the study and provided a questionnaire. Genetic counselling was delivered at a one-hour ‘in person’ appointment in private consulting rooms in West Leederville, Perth, Western Australia, and if genetic testing was performed, a further follow-up appointment was arranged to discuss the results and associated implications. Genetic counselling was provided by a single GC (SOS). Genetic counselling and testing were paid for by the participants. Participants paid a standard charge for the genetic counselling of AU$225 (US$165). The cost of genetic testing varied depending on whether single gene, predictive or multigene panel testing was conducted; the majority of participants had testing of a multigene panel at a cost of US$250 but some patients paid up to US$875. 

The GC (SOS) consults within a holistic women’s health group private practice with specialists in gynaecologic oncology, obstetrics and gynaecology, menopause medicine, clinical psychology, clinical sexology, physiotherapy and exercise physiology. The practice is located in a commercial office building of medical consulting rooms in West Leederville, Perth, Western Australia. Additional staff include a practice manager, two part-time receptionists and two part-time nurses. Patients are referred to the GC by medical specialists from both public hospital medical oncology, gynecologic oncology, breast and colorectal surgery outpatient clinics, and private consulting rooms, general practitioners, or by self-referral. Patients attending the practice are predominantly married, Australian women of European descent, over age 50 years, and with a personal or family history of breast, ovarian and colorectal cancer.

Genetic risk assessment was based on information relating to personal and family history of cancer, using risk scoring instruments such as BOADICEA. Written information about the study and the questionnaire was provided by reception staff. Informed consent was implicit in the completion and return of the questionnaire. Participants returned the questionnaires to the private practice in a self-addressed reply-paid envelope within one month. The questionnaires were stored in a locked cupboard of the offices of the Gynecological Cancer Research Group, St John of God Subiaco Hospital. Participants were not identifiable from the questionnaires. Data were extracted from questionnaires to an Excel spreadsheet and analysed by two investigators (CP and PC). The GC (SOS) was blinded to the study questionnaires and was not involved in data analysis. Ethical approval for the study was granted by The St John of God Healthcare Human Research Ethics Committee (Reference #976). The study was performed in accordance with the ethical standards as laid down in the 1964 Declaration of Helsinki and its later amendments.

*Data Analysis*

Data were analysed using IBM SPSS Statistics for Windows, version 26 (IBM Corp., Armonk, N.Y., USA). Categorical variables were described using frequency and percent, with missing data noted. Continuous scale variables were described using mean and standard deviation. Open text responses were tabulated but did not undergo thematic analysis. This was a non-comparative, single arm study conducted over three years. The sample size was pragmatic. 

## Results

*Participant Characteristics*

Seventy-five of 220 questionnaires were returned giving a response rate of 34%. Two of 75 questionnaires were incomplete and excluded from the analysis. No follow-up appointment occurred before participants received the survey. The participant characteristics are shown in Table 1. Forty-nine (67%) participants were referred by a medical specialist, eleven (15%) self-referred, nine (12%) by a general practitioner. Fifty-three participants were >50 years old and 95.9% were female. A majority of participants were married (72.6%) and of European ancestry (79.5%) with more than half having a university or postgraduate degree. Fifty of 73 participants (68.5%) had a personal history of cancer, 63 (86.3%) had a family history of cancer, and 43 (58.9%) had both a personal and family history of cancer (Table 2). Sixty-one participants (83.6%) underwent genetic testing following counselling (Table 1). All patients expressed a strong ‘yes’ or ‘no’ regarding genetic testing following pre-test counselling.

*Satisfaction with Genetic Counselling*

**Figure 1:**
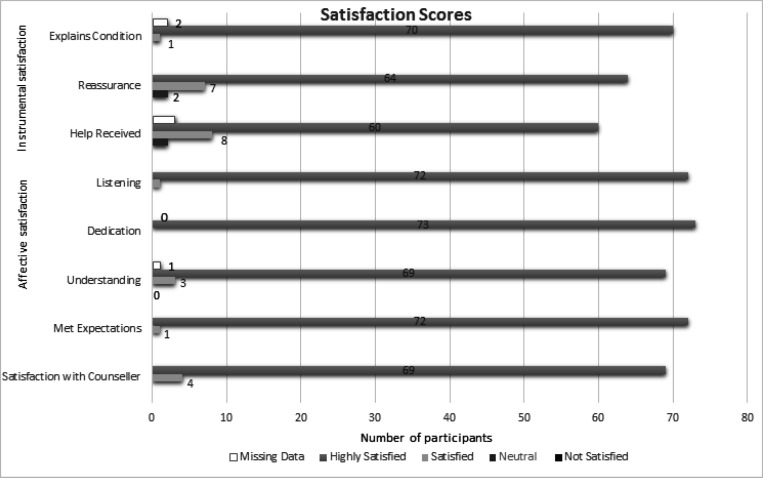
Elements Contributing to Patient Satisfaction with the Genetic Counsellor

Overall, 70 (96%) participants were highly satisfied with the genetic counsellor’s explanation; 64 (88%) were highly satisfied and seven (10%) were satisfied with the reassurance provided; 60 (82%) were highly satisfied and eight (11%) were satisfied with the help received; 72 (99%) participants had their expectations met and 69 (95%) participants were highly satisfied with the GC (Figure 1). Two participants indicated no change in reassurance following the service. Another two participants also indicated a neutral level of help provided by the service (Figure 1). The mean scores for the SCS Instrumental and Affective sub-scales are shown in Figure 1 and the mean scores for the SCS Procedural sub-scale in Table 3. 

*Procedural Satisfaction and ‘Value for Money’*

Other factors that affect participants’ satisfaction with the service include waiting time for the first appointment, length of time in the waiting room and treatment by other staff members. The majority of participants were highly satisfied with the service provided by other staff members and with the waiting times (Table 3). Sixty-eight participants (93%) were highly satisfied or satisfied with ‘value for money’ (Table 3).

*Open text responses*

Twenty-five respondents provided open-text responses and most described genetic counselling. Quotes are provided in the online supplementary file. Overall satisfaction was high for all those quoted.

**Table 1:** Participant Characteristics, Source of Referral and Whether Proceeded to Genetic Testing

	N (%)	
Age	<50 years	33 (45.2)
	>50 years	39 (53.4)
	Missing data	1 (1.4)
Gender	Female	70 (95.9)
	Male	3 (4.1)
Marital Status	Single	9 (12.3)
	De facto	10 (13.7)
	Married	53 (72.6)
	Missing data	1 (1.4)
Children	Yes	58 (79.5)
	No	15 (20.5)
Ethnicity	European	58 (79.5)
	Asian	5 (6.8)
	Other	3 (4.1)
	Missing data	7 (9.6)
Place of Birth	Australia	43 (58.9)
	Other	27 (37)
	Missing data	3 (4.1)
Language spoken	English as first language	63 (86.3)
	Other first language	7 (9.6)
	Missing data	3 (4.1)
Education	High School	31 (42.5)
	University	26 (35.6)
	Postgraduate degree	13 (17.8)
	Missing data	3 (4.1)
Referral source	Self-referred	11 (15.1)
	General Practitioner	9 (12.3)
	Medical specialist	49 (67.1)
	Missing data	4 (5.5)
Proceeded to genetic testing	Yes	61 (83.6)
	No	3 (4.1)
	Missing data	9 (12.3)

**Table 2:** Personal and Family History of Cancer in Participants

	Family history of cancer Yes	Family history of cancer No	Family history of cancer Not recorded	Total
	N	N	N	N
Personal history of cancer				
Yes	43	7	0	50
Personal history of cancer				
No	19	0	0	19
Personal history of cancer				
Not recorded	1	0	3	4
Total	63	7	3	73

**Table 3:** Procedural Satisfaction Scores

	Not satisfied	Neutral	Satisfied	Highly Satisfied	Missing Data	Mean Score (SD)
Waiting time prior to first appointment	1	2	11	56	3	3.74 (.582)
Length of time in waiting room	1	0	5	65	2	3.89 (.433)
Treatment by other staff members	0	0	3	70	0	3.96 (.200)
Overall satisfaction	0	0	3	70	0	3.96 (.200)
Value for money	0	3	10	58	2	3.77 (.513)

## Discussion

To our knowledge this is the first study to assess patient satisfaction with private genetic counselling for familial cancer in an Australian setting. Overall, we found that participants were extremely satisfied with the referral process and their genetic counselling. The majority also perceived the cost of private genetic counselling to be good value. 

Studies of patient satisfaction with public genetic counselling services have shown similar results to the current study. This suggests that satisfaction with private genetic counselling is comparable to public genetic services. One study of 156, predominantly white, married, female patients who had received counselling from a cancer genetics professional at the Penn State Cancer Institute, Philadelphia, USA, found that 96.1 % of patients were satisfied, very satisfied or extremely satisfied with their genetic counselling (Kausmeyer et al., 2006). Our findings are consistent with those of another study that used the Satisfaction with Genetic Counselling Scale and demonstrated a mean score between 9.9-11.7 (maximum score of 12) for instrumental, affective and procedural satisfaction combined, and for the three individual items - satisfaction with the information, met expectations, and overall satisfaction with the counselling - between 3.7-3.9 (maximum 4) (Nordin et al., 2002). In the current study the mean scores for satisfaction with the information, met expectations, and overall satisfaction with the counselling were 3.99, 3.99 and 3.95, respectively. Most participants were highly satisfied with the GC’s reassurance (mean score 3.85), listening skills (mean score 3.99), dedication (mean score 4.00) and understanding (mean score 3.96). 

Most participants were satisfied or highly satisfied with administrative procedures including the waiting time before the first appointment, length of time in waiting room and treatment by other staff members. Two scores of ‘one’ and 7 scores of ‘two’ related to aspects of procedural satisfaction, most commonly length of time prior to first contact, or to the patient’s health status and perception of their diagnosis and prognosis, suggesting that factors outside of the GC’s control may have influenced satisfaction. 

It was notable that most participants perceived private genetic counselling to be good value for money. Studies of patient-perceived value for money in genetic counselling are limited and this gap in knowledge represents an important area for future research. In women with ovarian cancer in whom genetic testing is recommended, the cost of testing was the most important factor, followed by probability of detecting a pathogenic variant, in 94 enrolled subjects recruited in a choice experiment survey of whom 68 (76.4%) presented for genetic counselling (Davidson et al., 2019). As current testing costs are now lower than those at the time this study was undertaken and some private patients are now eligible for Medicare-funded genetic testing, it could be hypothesized that satisfaction/perceived value would now be even higher.

*Study Limitations*

The study was conducted at a private practice in Western Australia with a single GC and included few male participants. Further, the population was highly educated, of mainly European ancestry, and although socio-economic data were not collected, participants self-funded the costs of genetic counselling and testing. Hence our findings may not be applicable in other clinical settings. There may have been response bias as we were not able to assess satisfaction in non-responders. Further, we did not collect demographic data in non-responders, so we were unable to assess whether responders differed from non-responders in age, gender, ancestry and personal and/or family cancer history. Only satisfaction with pre-test counselling was investigated and so we cannot make inferences about patient satisfaction with post-test counselling. Perception of value for money is likely to depend on several factors including available alternatives, image of the practice, trust, service quality, costs and consumer/patient factors including socio-economic group, income, and background. Our aim was to assess perceived value of genetic counselling, which had a standard charge, but it is conceivable that the variable cost of genetic testing may have biased this outcome in patients who proceeded to genetic testing.

In summary, this study shows a high level of self-reported patient satisfaction with a genetic counselling service at a private practice in Perth, Western Australia. Our findings support the value of this model of genetic counselling service delivery that is relatively new to Australia. Barriers to continuing to provide a high quality private genetic counselling service include low numbers of GCs in private practice and costs of genetic testing. Further research should investigate satisfaction with different models of private genetic counselling including telephone and video consultations for patients in regional and remote locations, and assessment of post-test counselling. Qualitative interviewing of patients may provide insight into any reported dissatisfaction, leading to implementation of changes that increase satisfaction with private genetic counselling further.

*List of Abbreviations*

BOADICEA - Breast and Ovarian Analysis of Disease Incidence and Carrier Estimation Algorithm 

BRCA - Breast Cancer Gene

FCC - Familial Cancer Clinic

GC - Genetic Counsellor

GSWA - Genetic Services of Western Australia

SCS - Satisfaction with Genetic Counselling Scale

WA - Western Australia

## Author Contribution Statement

CP: data acquisition, data analysis and interpretation, writing the first draft of the manuscript and final approval of the version to be published. Accountable for all aspects of the work and for the accuracy or integrity of the work. 

SOS: conception and design of the work, critical review of the manuscript and final approval of the version to be published. Accountable for all aspects of the work and for the accuracy or integrity of the work. 

NP: conception and design of the work, critical review of the manuscript and final approval of the version to be published. Accountable for all aspects of the work and for the accuracy or integrity of the work. 

JT: conception and design of the work; critical review of the manuscript and final approval of the version to be published. Accountable for all aspects of the work and for the accuracy or integrity of the work. 

PC: conception and design of the work; analysis and interpretation of data; drafting the manuscript and final approval of the version to be published. Accountable for all aspects of the work and for the accuracy or integrity of the work. 

Charmi N. Perera and Paul A. Cohen confirm that they had full access to all the data in the study and take responsibility for the integrity of the data and the accuracy of the data analysis. All of the authors gave final approval of this version to be published and agree to be accountable for all aspects of the work in ensuring that questions related to the accuracy or integrity of any part of the work are appropriately investigated and resolved.
